# Nanoparticles Carrying Conserved Regions of Influenza A Hemagglutinin, Nucleoprotein, and M2 Protein Elicit a Strong Humoral and T Cell Immune Response and Protect Animals from Infection

**DOI:** 10.3390/molecules28186441

**Published:** 2023-09-05

**Authors:** Anna A. Zykova, Elena A. Blokhina, Liudmila A. Stepanova, Marina A. Shuklina, Olga O. Ozhereleva, Liudmila M. Tsybalova, Victor V. Kuprianov, Nikolai V. Ravin

**Affiliations:** 1 Institute of Bioengineering, Research Center of Biotechnology of the Russian Academy of Sciences, Moscow 119071, Russia; 2 Smorodintsev Research Institute of Influenza, Russian Ministry of Health, St. Petersburg 197376, Russia

**Keywords:** nanoparticle, self-assembling peptide, influenza A, M2e peptide, hemagglutinin, immune response

## Abstract

Current influenza vaccines are mainly strain-specific and have limited efficacy in preventing new influenza A strains. Efficient control of infection can potentially be achieved through the development of broad-spectrum vaccines based on conserved antigens. A combination of several such antigens, including the conserved region of the second subunit of the hemagglutinin (HA2), the extracellular domain of the M2 protein (M2e), and epitopes of nucleoprotein (NP), which together can elicit an antibody- and cell-mediated immune response, would be preferred for vaccine development. In this study, we obtained recombinant virus-like particles formed by an artificial self-assembling peptide (SAP) carrying two epitopes from NP, tandem copies of M2e and HA2 peptides, along with a T helper Pan DR-binding epitope (PADRE). Fusion proteins expressed in *Escherichia coli* self-assembled in vitro into spherical particles with a size of 15–35 nm. Immunization of mice with these particles induced strong humoral immune response against M2e and the entire virus, and lead to the formation of cytokine-secreting antigen-specific CD4+ and CD8+ effector memory T cells. Immunization provided high protection of mice against the lethal challenge with the influenza A virus. Our results show that SAP-based nanoparticles carrying conserved peptides from M2, HA, and NP proteins of the influenza A virus, as well as T helper epitope PADRE, can be used for the development of universal flu vaccines.

## 1. Introduction

The influenza A virus is one of the most common respiratory pathogens affecting humans and animals. The main problem in the prevention of this disease is the high variability of influenza viruses as a result of antigenic shifts and rapid antigenic drift. As a result, it is necessary to update the composition of traditional influenza vaccines every year in accordance with the circulating strains of the virus. The emergence of new seasonal and pandemic strains of the influenza A virus promotes the development of influenza vaccines that can induce a broad protective immune response. One of the most promising strategies in the development of such vaccines is the use of conservative viral antigens to generate a broad spectrum of cross-reactive antibodies as well as a potent T cell response. The extracellular domain of the transmembrane protein M2 and conserved regions in hemagglutinin (HA) and nucleoprotein (NP) may be promising antigens for this purpose [1,2].

M2e is a highly conserved peptide. However, M2e is a poor immunogen, so the use of carriers and/or adjuvants to enhance immune responses is necessary. Various types of M2e-based recombinant vaccines can provide broad-spectrum protection in animal models [3,4]. M2e-specific antibodies, mainly IgG, are major players in the immune defense. Anti-M2e antibodies do not have virus-neutralizing activity but have been shown to confer protection through the mechanism of antibody-dependent cell-mediated cytotoxicity (ADCC) of infected cells, limiting viral replication further and promoting accelerated clearance of the virus as well as reducing the severity of the disease [5,6].

Hemagglutinin also has conserved sequences in the stem region of the second subunit (HA2). Such fragments can induce the formation of antibodies against a wide range of hemagglutinins of different subtypes [7]. It is known that vaccine candidates based on recombinant proteins, including M2e and HA2 epitopes, are highly immunogenic and have protective properties [8,9]. Antibodies specific to the HA2 stem region are not necessarily neutralizing and may instead use other mechanisms that promote broad protection, such as ADCC, to clear the virus after infection [10,11,12]. 

Another promising antigen for universal vaccine development may be conserved fragments of nucleoprotein (NP). The NP protein is relatively well conserved in influenza A viruses, with a difference in amino acid sequences of less than 11% [13]. It is a target for the cytotoxic T-lymphocyte (CTL) cross-reactive response to influenza A viruses [14,15]. CTL induction is important for recovery from influenza infection by killing virus-infected cells [16]. Due to sequence conservatism and the induction of broad NP-specific immunity, NP is attractive for the development of a universal influenza vaccine. NP fragments have been used in several universal vaccine candidates at different stages of clinical trials (CT phase III: Multimeric-001 (M-001), phase II: FLU-v, phase I: FP-01.1) (according to the database Universal Influenza Vaccine Technology Landscape). In the Multimeric-001 recombinant vaccine, three NP protein epitopes were included: a Th epitope and 2 CTL epitopes (335–350 a.a. and 380–393 a.a.). The FLU-v peptide vaccine contains the NP protein consensus sequence for influenza A (255–275 a.a.) and B (307–325 a.a.) viruses [17]. The safety and efficacy of these vaccines, as well as their ability to induce an NP-specific T cell response, have been shown [18,19]. 

However, the task of developing highly immunogenic vaccines based on conservative influenza antigens is far from being solved, and the candidate vaccines being developed are still inferior to traditional strain-specific vaccines in terms of their effectiveness. One way to increase the immunogenicity of short peptide antigens is to attach them to nanosized particles that are formed by viral capsid proteins or artificial self-aggregating peptides, SAP [20,21]. Self-aggregating peptides under certain conditions can form corpuscular particles similar to virus-like particles (VLPs); however, the mechanism of their assembly seems to be simpler than the assembly of VLPs from capsid proteins. SAPs have already been successfully used for epitope presentation resulting in high immunogenicity [22,23,24,25].

Previously, as a basis for the creation of VLPs we used SAP, consisting of 63 a.a., where N-terminal 36 a.a. represent a modified pentameric helical domain, and the C-terminal 25 a.a. correspond to the trimeric helical domain [26,27]. The two domains are connected by two glycine residues and contain two cysteines, which form intramolecular disulfide bridges for interaction of these domains. Previously, we obtained recombinant mosaic proteins containing tandem copies of M2e and stalk HA epitopes fused to SAP [27]. We found that SAP particles carrying both M2e and HA2 induced potent immune responses and provided high protection of mice against the lethal challenge with different subtypes of the influenza A virus [27].

**Figure 1:**
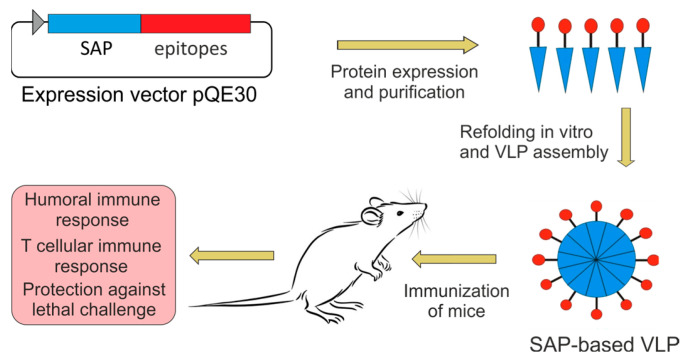
Overall experimental design and workflow.

A combination of multiple conserved antigens that together can elicit an antibody- and cell-mediated immune response against a broad spectrum of influenza viruses would be preferred for the “universal” vaccine’s development. To further stimulate the T cell’s immune response, the recombinant proteins could include the T helper epitope PADRE [28]. In this study, we designed, produced, and characterized recombinant SAP-based nanoparticles carrying conserved influenza antigens, namely the M2e peptide, the conserved HA2 fragment, and NP epitopes as well as the T helper epitope PADRE (Figure 1). We found that such nanoparticles induce an effective humoral and T cell immune response and protect immunized mice from lethal influenza infection.

## 2. Results

### 2.1. Design of Recombinant Proteins

The M2e peptide, the fragment of the second subunit of HA (76–130 a.a.), and two epitopes from the nucleoprotein (255–275 a.a and 335–350 a.a.) were used as conserved antigens for the development of new influenza vaccine candidates. Since an increase in the copy number of the M2e peptides in the recombinant protein enhances the immune response against M2e [29], we included four copies of M2e in the fusion protein. For the same purpose, two copies of the HA2 peptide were included. The formation of nanosized particles should have provided by a self-aggregating peptide, SAP. Two nucleoprotein epitopes and a T helper epitope PADRE flanked by dilysin linkers were placed at the N-terminus of the SAP. Four copies of the M2e and two copies of HA2 were linked to the C-terminus of SAP. These peptides were separated from the C-terminus of SAP by a rigid helical linker SP for more efficient exposure of the peptides to the surface of the nanoparticles by increasing the length of the helical domain of SAP. The two copies of the HA2 peptides were separated from each other by a short flexible 10 a.a. long glycine linker. The control protein had a similar design but did not contain SAP. 

Six histidine residues encoded by the expression vector pQE30 appeared at the N-terminus of the recombinant proteins to enable their purification by metal affinity chromatography. This hexahistidine tag was separated from the rest of the recombinant protein by a flexible 19 a.a. long glycine/serine-rich linker (19S) to facilitate the interaction of proteins with Ni-NTA sorbent.

**Figure 2:**
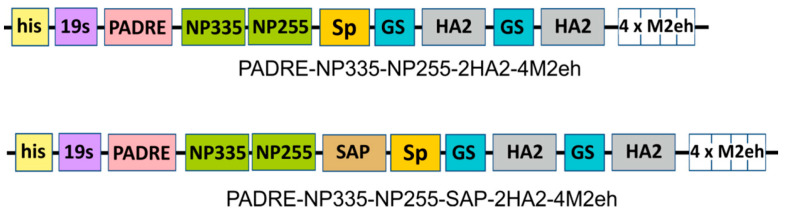
Structure of the recombinant proteins.

In total, two recombinant fusion proteins were designed for expression in *E. coli*: PADRE-NP335-NP255-SAP-2HA2-4M2eh aimed to form nanoparticles and the control protein PADRE-NP335-NP255-2HA2-4M2eh without SAP (Figure 2).

### 2.2. Expression and Purification of Recombinant Proteins

**Figure 3:**
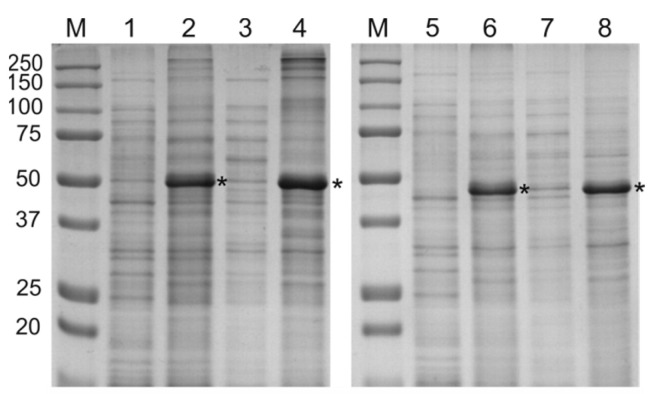
Expression of the recombinant proteins in *E. coli*. The proteins were analyzed using SDS-PAGE. M, molecular weight marker (sizes are shown in kDa). Lanes 1–4, proteins from *E. coli* strain producing PADRE-NP335-NP255-SAP-2HA2-4M2eh; lanes 5–8, proteins from *E. coli* strain producing PADRE-NP335-NP255- 2HA2-4M2eh. Lanes 1 and 5, proteins isolated before induction. Lanes 2 and 6, total protein samples isolated after induction. Lanes 3 and 7, proteins from soluble fraction after induction. Lanes 4 and 8, proteins from insoluble fraction after induction. The positions of the target proteins are shown with asterisks (*).

The fusion genes were cloned in vector pQE30 and expressed in *E. coli*. Both recombinant proteins were successfully expressed at levels of about 10–15% of the total protein but appeared to be almost completely insoluble (Figure 3).

**Figure 4:**
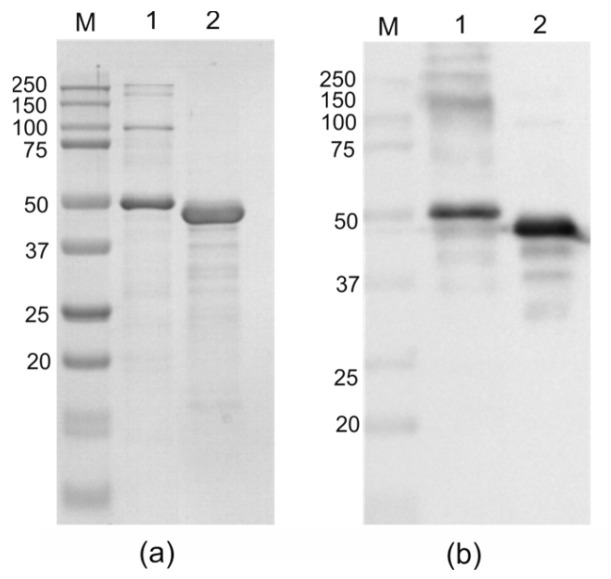
Analysis of the purified recombinant proteins by SDS–PAGE (**a**) and Western blotting (**b**). M, molecular weight marker (sizes are shown in kDa). Lanes 1, 2—purified proteins PADRE-NP335-NP255-SAP-2HA2-4M2eh and PADRE-NP335-NP255-2HA2-4M2eh, respectively. Polyclonal antibodies against M2eh were used for Western blotting.

Recombinant proteins were purified by nickel affinity chromatography under denaturing conditions (Figure 4). For correct refolding of proteins, they were then subjected to stepwise dialysis with a decrease in urea concentration. Proteins remained soluble in PBS after dialysis and were recognized by antibodies against M2e in Western blot (Figure 4). The calculated molecular weight of the protein PADRE-NP335-NP255-SAP-2HA2-4M2eh is 47 kD; in addition to the monomeric form, it formed multimers migrating in SDS-PAGE in the region from 100 to 250 kDa. Aggregates were not observed in the case of the control protein without SAP (Figure 4). 

### 2.3. Formation of Nanoparticles

**Figure 5:**
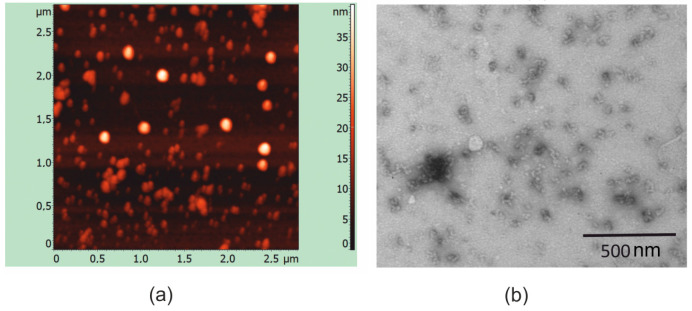
Analysis of the structure of PADRE-NP335-NP255-SAP-2HA2-4M2eh nanoparticles using atomic force microscopy (**a**) and electron microscopy (**b**).

The assembly of purified and refolded recombinant proteins into nanosized structures was examined using atomic force and electron microscopy. The protein PADRE-NP335-NP255-SAP-2HA2-4M2eh has been found to self-assemble into spherical nanoparticles ranging in size from 15 to 35 nm (Figure 5). In contrast, no particulate structures have been observed in case of protein without SAP.

### 2.4. Humoral Immune Response

To characterize the immunogenicity of the obtained proteins, Balb/c mice were immunized subcutaneously with nanoparticles formed by the recombinant protein with the SAP peptide and with the control protein unable to form nanoparticles. The sera and BAL fluids were collected after the third immunization and analyzed using ELISA to identify IgG antibodies recognizing M2e and the influenza virus A/Aichi/2/68 (H3N2).

**Figure 6:**
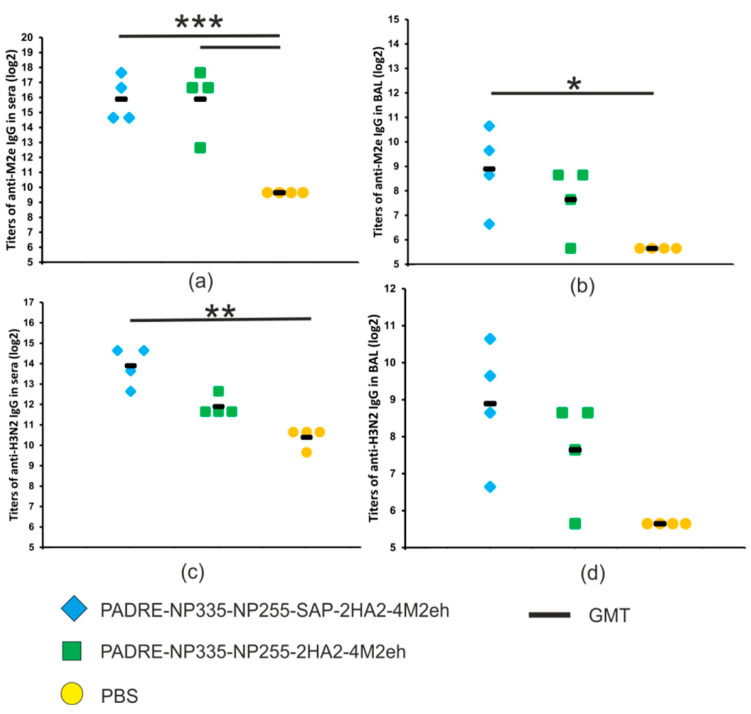
Antibody response in sera and BAL of immunized mice. Titers of anti-M2e IgG in sera (**a**) and BAL (**b**). Titers of IgG antibodies recognizing A/Aichi/2/68 (H3N2) virus in sera (**c**) and BAL (**d**). Data are presented as the log2 of geometric mean titers (GMT) and values observed in individual mice. The *p* values between groups are indicated (*** 0.0001 < *p* < 0.001, ** 0.001 < *p* < 0.01, * 0.01 < *p* < 0.05).

A strong M2e-specific immune response developed in both experimental groups of immunized mice: anti-M2e IgG titers in sera were significantly higher than in the control group injected with PBS (*p* < 0.001). There were no significant differences in serum anti-M2e IgG titers between the two experimental groups (Figure 6a). By contrast, the titers of anti-M2e IgG in BAL fluid in mice immunized with PADRE-NP335-NP255-SAP-2HA2-4M2eh nanoparticles were significantly higher than in the groups of mice immunized with the control protein lacking SAP (Figure 6b).

Since in addition to M2e the recombinant proteins contained conserved HA fragments, we tested the formation of virus-specific antibodies. IgG antibodies in sera and BAL recognized influenza virus A/Aichi/2/68 (H3N2) from phylogenetic group 2, and virus-specific antibody titers were significantly higher in mice immunized with PADRE-NP335-NP255-SAP-2HA2-4M2eh protein than in all other groups (Figure 6b,c). Therefore, the presence of the SAP peptide promoting the formation of nanoparticles is important for the induction of virus-specific humoral immune response.

### 2.5. T Cellular Immune Response

To study the formation of the T cell response in mice, the PADRE-NP335-NP255-SAP-2HA2-4M2eh protein was chosen, since it caused the induction of not only the M2e-specific, but also a strong virus-specific immune response. T lymphocytes were isolated from the spleen of immunized and control (PBS) mice two weeks after the last immunization and assayed for activation by M2e, NP255/NP335 peptides, and the A/Aichi/2/68 (H3N2) virus using intracellular cytokine staining.

**Figure 7:**
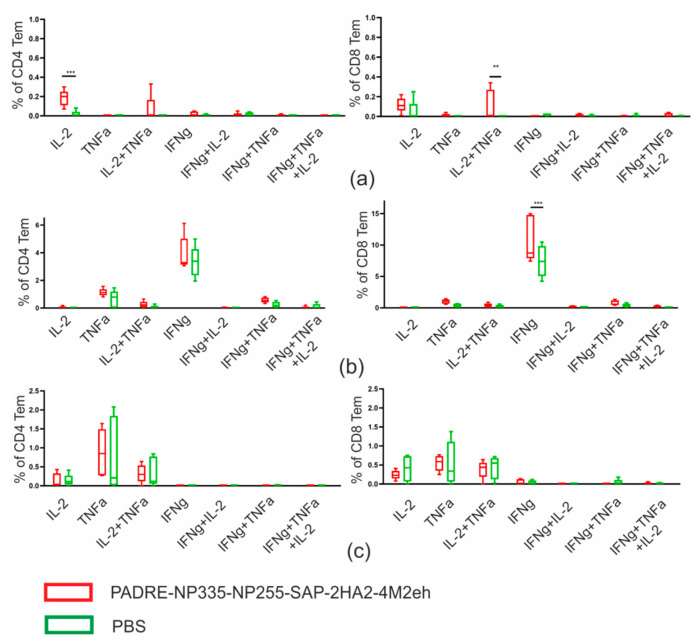
Antigen-specific T cell response in the spleen of immunized mice. T cells were stimulated with M2e (**a**) or with A/Aichi/2/68 (H3N2) virus (**b**) or with NP peptides (**c**). The data are presented in the form of a Tukey plots. The *p* values between groups are indicated (*** *p* < 0.001, ** *p* < 0.01).

Recombinant protein induced the formation of M2e-specific and virus-specific effector memory (Tem) CD4+ and CD8+ T cells (Table 1), while the measured fractions of NP-specific CD4+ and CD8+ Tem cells were nearly identical between experimental and control groups. Then, we analyzed the ability of antigen-specific T cells to produce the cytokines IL-2, TNF-α, and IFN-γ (Figure 7). 

The population of M2e-spesific CD4+ Tem cells was predominantly IL-2 single producers (0.18%) and double-producing IL-2/TNF-α (0.07%) cells. The cytokine-producing M2e-specific CD8+ Tem cells were mostly represented by IL-2/TNF-α double producers and (0.11%) and IL-2 single producers (0.12%). The population of H3N2-specific CD8+ Tem cells was represented by IFN-γ producers (10.8%).

**Figure 8:**
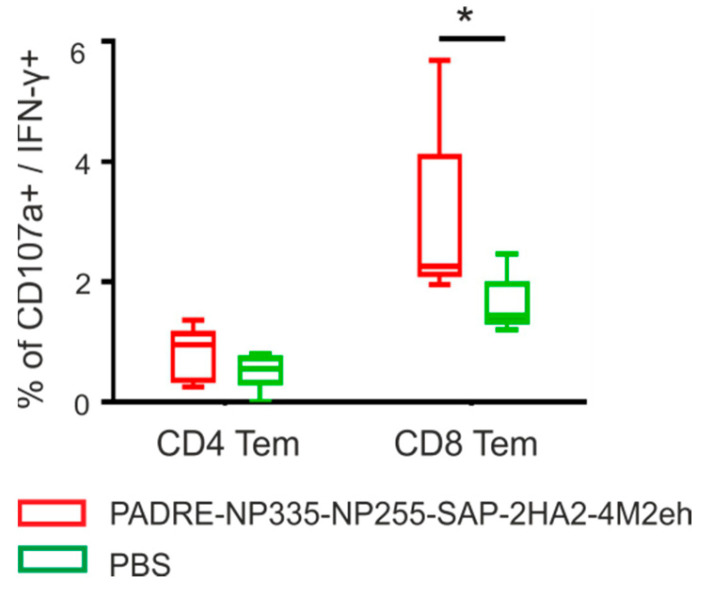
Expression of CD107a degranulation marker on virus-stimulated Tem cells. The data is presented in the form of a Tukey plots. The *p* values between groups are indicated (* 0.01 < *p* < 0.05).

One of the ways to evaluate the cytotoxic function in cells is to determine the expression of the membrane glycoprotein CD107a. The emergence of virus-specific polyfunctional CD8+ Tem cells expressing CD107a and IFN-γ+ (2.9%) was detected (Figure 8).

### 2.6. Protection against Lethal Influenza Challenge

**Figure 9:**
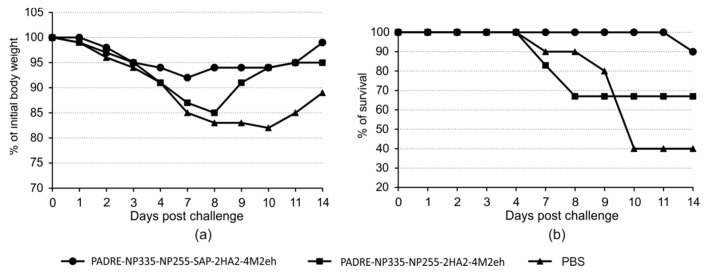
Protective efficacy of recombinant proteins. Body weight (**a**) and survival rate (**b**) were monitored daily for 14 days post-challenge.

To assess the protective efficacy of the obtained recombinant proteins, immunized and control mice were infected with the influenza A/Aichi/2/68 (H3N2) strain at a dose of four LD_50_. Mice immunized with the PADRE-NP335-NP255-SAP-2HA2-4M2eh protein, forming nanoparticles, showed 90% survival and minimal body weight loss (8%). In comparison, in mice immunized with a protein PADRE-NP335-NP255-2HA2-4M2eh without SAP, the survival after lethal challenge was 67%. A more pronounced weight loss (up to 15%) and slower recovery of mice were observed (Figure 9).

## 3. Discussion

Currently, there are a number of studies devoted to the creation of virus-like particles and their use for the development of recombinant vaccines. Compared to traditional attenuated and inactivated vaccines against viral diseases, VLP-based vaccines do not contain the viral genome and represent a safer alternative. Moreover, they provide an opportunity for presentation of multiple copies of target antigens. Such repetitive antigenic display can greatly increase the immunogenicity of short peptides and epitopes [30]. However, in many cases, foreign inserts prevent the assembly of VLPs formed by viral capsid proteins. An alternative building block for VLPs could be artificial self-aggregating peptides (SAPs). The particles formed from SAP can also be considered VLPs since they also have a certain structure and can expose target antigens on the surface [31].

The inclusion of different antigens of the influenza virus could expand the spectrum of inducible immunity provided by new recombinant vaccines. Therefore, several epitopes enabling the induction of different mechanisms of immune response, not only humoral but also T cellular, were used in this study. Two variants of recombinant proteins based on human influenza A virus antigens were designed and produced. Conserved sequences of the extracellular domain of the M2 protein (M2e) and the second subunit of the hemagglutinin, as well as two conserved peptides of the nucleoprotein, were used. The Pan DR-binding epitope (PADRE) was also included in the recombinant proteins. This epitope can activate antigen-specific CD4+ T cells, simultaneously initiating the innate immune response [28]. 

The self-aggregating peptide SAP was used as a building block for assembly of recombinant protein into virus-like nanoparticles. Its alpha-helical sequences, due to coiled-oil interactions, can form nanoparticles that can serve for antigen presentation to the immune system [26]. The HA2 and M2e peptides were inserted after the trimeric SAP oligomerization domain. At the same time, the number of copies of these antigens was increased: two copies of HA2 and four copies of M2e were inserted. It was previously shown that an increase in the copy number of the M2e peptide from one to four in the HBc antigen, which also forms VLP, enhances the immune response against M2e [29]. The NP peptides and the PADRE epitope were located at the N-terminus of the pentameric domain of the SAP. As a result, recombinant SAP-based particles presented multiple copies of different conserved influenza virus antigens sequences. Due to such a structure, recombinant particles appeared to be highly immunogenic and capable of inducing both humoral and T cellular immune response.

Recombinant proteins were expressed in *E. coli* cells and appeared to be completely insoluble, which can be explained by the many hydrophobic amino acids in the PADRE, NP peptides, and HA2. Purification of recombinant proteins was carried out under denaturing conditions, followed by stepwise refolding, so that the proteins optimally refolded without the formation of chaotic intermolecular bonds. With this refolding method, it was possible to obtain both proteins in a soluble form. The protein with the SAP peptide self-assembled into 15–35 nm spherical nanoparticles.

After immunization, we observed no differences in titers of anti-M2e IgG in serum between the groups of mice immunized with recombinant proteins with and without the SAP peptide. A control protein that does not contain the SAP peptide comprised four tandem copies of M2e. Such linear multiepitope proteins can effectively induce an immune response [17]. Previous studies have shown that antibodies specific to M2e cannot directly neutralize the influenza virus but can provide cross-protection by inducing several mechanisms of immune responses mediated by antibodies and cells. The best characterized defense mechanisms are ADCC (antibody-dependent cell-mediated cytotoxicity), ADCP (antibody-dependent cellular phagocytosis), and CDC (complement-dependent cytotoxicity) [32]. Therefore, antibodies to M2e are an important indicator of protection against influenza viruses.

In contrast, IgG titers to the entire A/Aichi/2/68 (H3N2) virus were significantly higher in sera of mice immunized with the SAP-carrying protein than in the group of mice immunized with the similar protein without SAP. The weak immune response to the virus in the case of PADRE-NP335-NP255-SAP-2HA2-4M2eh is likely due to its inability to form nanoparticles. The immune response to the whole influenza virus reflects predominantly the production of antibodies to the HA2 peptide from the stem region of HA. The activity of such antibodies can lead to the neutralization of influenza viruses, although their neutralizing activity is weaker than that of the antibodies to the head domain of hemagglutinin, which directly interfere with binding to the cell receptor. Antibodies to the stem region of HA also provide a variety of indirect protection mechanisms, including antibody-dependent effector mechanisms [33] and inhibition of neuraminidase enzymatic activity [34,35]. Therefore, it is important to include conserved fragments of hemagglutinin in the candidate vaccines to form a broader immune response than in case of only M2e.

Currently, the main means of combating influenza are traditional inactivated and live attenuated vaccines, which induce virus-neutralizing antibodies targeting the hemagglutinin. However, they do not specially target the T cell immune response despite evidence that it plays a significant protective role during infection [36]. Moreover, most T cell epitopes are found in the internal proteins of the influenza virus, while current vaccines contain mainly HA and NA. In our work, we use CD8+ T cell epitopes from the nucleoprotein as well as the universal CD4+ T cell epitope PADRE to generate a broad T cell response.

The induction of strong long-term CD4+/CD8+ T cell immunity is an important tool for efficient protection [37]. Neither CD8+ cells, nor CD4+ cells, nor B cells alone can provide efficient virus clearance [38]. Therefore, the immune response to the influenza virus requires a complex interaction between cytotoxic T cells, antibody-producing B cells, and helper T lymphocytes. Mouse models of influenza A pneumonia are a well-established experimental system for studying T cell-mediated immunity. In particular, the T cell immune response to the influenza infection has been well characterized in C57BL/6 (B6,H2b) mice [39]. It was on this line of mice that we studied the formation of the T cell response after immunization with PADRE-NP335-NP255-SAP-2HA2-4M2eh nanoparticles. The formation of CD4+ Tem cells in the spleen, which produced IL-2 upon stimulation with M2e, as well as the formation of M2e-specific CD8+ Tem cells producing two types of cytokines, TNF-α and IL-2, was observed. It was also found that immunization caused the formation of virus-specific CD8+ producers of IFN-γ. CD8+ T cells, in addition to their cytotoxic function, can secrete chemokines that are able to attract other immune cells. The production of cytokines by CD8+ T cells, such as IFN-γ, TNF-α, further enhances their cytotoxicity and modulates the innate and adaptive immune systems, directing immune responses towards the Th1 pathway [40]. Overall, nanoparticles bearing conserved influenza virus antigens and the PADRE epitope induced the formation of antigen-specific multifunctional CD4+/CD8+ effector memory T cells.

The study of T cell response showed the emergence of virus-specific polyfunctional CD8+ memory T cells expressing CD107a and IFN-γ in the spleen of immunized mice. CD107a belongs to the LAMP family and are highly glycosylated membrane proteins of lytic granules containing granzyme and perforin [41]. CD107a expression has been described to be strongly upregulated on the surface of CD8+ T cells upon activation of the cytotoxic function of these cells by releasing the contents of such granules. It can be proposed that CD8+ T cells expressing CD107a and IFN-γ exhibit a cytotoxic function to the influenza virus.

Previously, we obtained similar SAP-based nanoparticles bearing two copies of HA2 and four copies of M2e on the surface [27]. The incorporation of PADRE and NP epitopes into these nanoparticles expanded the spectrum of the T cell immune response. Previously obtained particles caused the formation of CD4+ Tem cells in the spleen that produced cytokines upon stimulation with M2e. Those M2e-specific CD4+ Tem cells were single- (IFN-γ) and double-producing (IL-2/TNF-α) cells [27]. The present study also identified CD4+ Tem cells producing IL-2 and TNF-α, but most cells were single producers of IL-2. More importantly, immunization of mice with the previously described nanoparticles did not induce the formation of M2e-specific CD8+ Tem and virus-specific CD4+ and CD8+ Tem cells at a level significantly different from the PBS control [27]. Here we observed both the formation of M2e-specific CD8+ Tem cells producing IL-2 and TNF-α and the generation of virus-specific CD4+ and CD8+ Tem cells producing IFN-γ. Since T cell immunity is important for broad protection against influenza infections [42,43], expanding the spectrum of T cell response should increase the effectiveness of the vaccine candidate.

Although immunization with our nanoparticles induced the formation of M2e-specific and virus-specific CD4+ and CD8+ Tem cells, the formation of NP-specific Tem cells was not observed. It is possible that it was caused by the location of the NP epitopes on the nanoparticle, which was not optimal for generating an immune response because, unlike HA2 and M2e, the NP epitopes were attached to the N-terminus of the SAP peptide. A possible solution to this problem would be to use a mixture of three types of SAP particles carrying only one of the antigens (NP epitopes, HA2, and M2e). An alternative would be to obtain mosaic nanoparticles by self-assembly of three SAP-based recombinant proteins in vitro.

Traditional inactivated and live attenuated vaccines are aimed at neutralizing the influenza virus and thus preventing infection, while recombinant vaccines based on conserved viral antigens contribute to the destruction of infected cells and accelerated elimination of the virus: they are aimed at reducing the duration of the disease and preventing heavy forms. The obtained recombinant proteins that self-assemble into nanoparticles were able to provide protection against a lethal dose of the influenza A/Aichi/2/68 (H3N2) virus. Mice immunized with this drug showed a 90% survival rate after infection with the influenza A virus. The mice had a slight loss of body weight, and they began to recover on the seventh day after infection. In contrast, in the case of the protein without the SAP peptide 19S-PADRE-NP335-NP255-Sp-2(GS_HA2)-4M2eh, less effective protection and greater body weight loss were observed, which led to a long recovery. These data further support the importance of the formation of nanoparticles for the effective development of an immune response against conserved influenza virus antigens.

## 4. Materials and Methods

### 4.1. Components of SAP-Based Fusion Proteins

The following peptides were included in the fusion protein:

M2eh (SLLTEVETPIRNEWGSRSNDSSD)—consensus sequence of the extracellular domain of the M2 protein of the human influenza virus without the first methionine. In the M2e sequence, cysteines at positions 17 and 19 were replaced by serines to prevent the formation of disulfide bonds. It has been shown that this replacement does not affect the immunological characteristics of M2e [44]. 

HA2 (RIQDLEKYVE DTKIDLWSYN AELLVALENQ HTIDLTDSEM NKLFEKTRRQ LRENA)—consensus sequence of conserved region of HA2 (a.a. 76–130) of influenza A viruses A/H3N2 and A/H7N9 [9].

NP255 (DLIFLARSAL ILRGSVAHKS) and NP335 (SAAFEDLRVL SFIR GY)—epitopes from the nucleoprotein of the influenza A virus [45,46].

SAP (DMELRELQET LAALQDVREL LRQQVKQITF LKCLLMGGRL LCRLEELERR LEELERRLEE LER)—self-assembling peptide, modified as described earlier [27].

Sp (EAAAKEAAAKEAAAKEAAAKEAAAK)—rigid helical linker [47].

19s (GTSGSSGSGSGGSGSGGGG)—flexible glycine/serin-rich linker [48]. 

GS (GGGGSGGGGS)—short flexible linker.

PADRE (AKFVAAWTLKAAA)—universal T helper epitope, the pan HLA DR-binding epitope [28]. 

### 4.2. Genes Encoding Fusion Proteins and Expression Vectors

The previously constructed expression vector pQE30/19s_SAP_Sp_2(GS)HA2_ 4M2eh [27] was used as a basis for further modifications. This pQE-based plasmid contained a hybrid gene encoding the SAP peptide fused to 2 copies of HA2 and 4 copies of M2e. 

The nucleotide encoding PADRE-NP335-NP255 fusion was obtained by annealing of a pair of synthetic oligonucleotides and cloned into pQE30/19s_SAP_Sp_ 2(GS)HA2_4M2eh between 19s and the SAP. The resulting vector was designated as pQE30/SAP-PADRE-NP335-NP255-2HA2-4M2eh. Vector pQE30/PADRE- NP335-NP255- 2HA2-4M2eh for the expression of a control protein without the SAP was obtained by inserting the PADRE-NP335-NP255 sequence into the vector pQE30/19s_Sp_2(GS)HA2_ 4M2eh between 19s and Sp.

Both vectors encoded an N-terminal hexahistidine tag, the presence of which allowed protein purification by metal affinity chromatography.

### 4.3. Expression and Purification of Fusion Proteins

For the expression of recombinant proteins, the constructed vectors were transformed into *Escherichia coli* cells of the DLT1270 strain. The cultures were grown in LB medium at 37 °C with shaking to the middle of the logarithmic growth phase (OD_600_~0.5). Expression of recombinant proteins was induced by isopropyl β-D-1-thiogalactopyranoside (1 mM), and the cultures were grown overnight at 28 °C.

The cells were collected by centrifugation at 4000× *g* for 30 min and resuspended in 10 mM PBS (pH 7.2) containing 1 mM of lysozyme. After freezing and thawing, the cells were lysed by ultrasound on ice using Bandelin SONOPULS HD 2200 (Bandelin electronic GmbH, Berlin, Germany). After centrifugation at 4000× *g* for 30 min, the pellet was solubilized in denaturing buffer (10 mM phosphate buffer, 0.5 M NaCl, 6 M guanidine chloride) and incubated overnight at 4 °C. Then, this solution was subjected to centrifugation at 13,000× *g* for 10 min and the obtained supernatant was used for purification of recombinant proteins using metal-affinity chromatography.

His-tagged recombinant proteins were adsorbed on Ni-NTA agarose (Qiagen, Hilden, Germany) in 10 mM phosphate buffer with 0.5 M NaCl and 6 M guanidine chloride. The first washing was performed in the same buffer additionally containing 16 mM of imidazole. The washing was carried out in a denaturing buffer with urea (10 mM phosphate buffer, 6 M urea, 0.5 M NaCl, 16 mM imidazole). Adsorbed proteins were eluted with the same buffer containing 500 mM of imidazole. Then, the purified proteins were dialyzed against 10 mM PBS with a stepwise decrease in urea concentration: 6 M, 4 M, 2 M, 1 M. The final dialysis was carried out against 10 mM PBS (pH 7.2).

### 4.4. Western Blot Analysis of Protein Samples

After separation in SDS-PAGE, the proteins were transferred to the Hybond-P membrane (GE Healthcare, Chicago, IL, USA) by semi-dry transfer using the Trans-Blot Turbo Transfer System (Bio-Rad Laboratories, Hercules, CA, USA). To prevent non-specific binding of antibodies, the membrane was incubated in a 5% solution of skimmed dry milk in 10 mM PBS for 1 h with constant stirring. Then, the membrane was incubated with mouse anti-M2e polyclonal antibodies, washed, and then treated with the anti-mouse IgG HRP conjugate (W4021, Promega, Madison, WI, USA). Specific protein complexes were visualized using the Western Blot ECL Plus kit (GE Healthcare).

### 4.5. Structural Analysis of Recombinant Proteins

Samples of recombinant proteins after refolding were analyzed using atomic force microscopy and transmission electron microscopy. Atomic force microscopy was performed using an Integra Prima microscope (NT-MDT, Moscow, Russia) in a semi-contact mode using a 35 nm gold cantilever. Electron microscopy was performed using a JEM 1400 instrument (JEOL, Tokyo, Japan) using a method of negative contrasting with 1% uranyl acetate solution.

### 4.6. Immunization of Animals

Female BALB/c mice (16–18 g, 10–14 per group) were immunized subcutaneously 3 times at 2-week intervals with 50 µg of recombinant protein with a sodium desoxyribonucleate (Derinat, 1 mg) adjuvant. The control group was subcutaneously injected with PBS. Samples of sera and broncho–alveolar lavages (BALs) of four mice were collected after the third immunization to determine antibody titers.

To study the cellular immune response, female C57BL/6 mice (haplotype H-2b) (16–18 g, 5 mice in each group), were immunized in the same way. Fourteen days after the third immunization, the animals were euthanized in a CO_2_ chamber and spleen cell suspension samples were isolated.

### 4.7. Antibody Detection in the Sera and BAL by ELISA

Antibody titers in BAL and sera samples were measured using enzyme-linked immunosorbent assay (ELISA). The synthetic M2e peptide G37 (SLLTEVET PIRNEWGCRCNDSSD) or the influenza virus A/Aichi/2/68(H3N2) in PBS (pH 7.2) were used to coat 96-well microtiter plates (Greiner, Germany). Goat polyclonal anti-mouse IgG (Abcam, Cambridge, UK) labeled with a horseradish peroxidase was used as a conjugate. After adding tetramethylbenzidine substrate (BD Bioscience, Franklin Lakes, NJ, USA) and color development, the reaction was stopped by H_2_SO_4_, and OD_450_ was measured on a microplate spectrophotometer.

### 4.8. Isolation of Cells from the Spleen to Analyze the T Cellular Immune Response

The spleens of mice were aseptically removed, disintegrated with a Medimachine and Medicons Cod. 79,300 (BD Biosciences, San Diego, CA, USA), and purified from cellular debris by filtration through a 70 μm filter (Syringe Filcons, BD Biosciences). Then, the cell suspension was washed with RPMI-1640 complete medium containing 7.5% FBS, 0.05 mM mercaptoethanol and 2 mM L-glutamine. Erythrocytes were lysed by 3 mL of ACK buffer (1.0 M KHCO_3_, 0.15 M NH_4_Cl, 0.1 mM Na_2_EDTA, pH 7.2–7.4). Then, 10 mL of RPMI-1640 medium was added to the solution and the cells were collected by centrifugation at 500× *g* for 5 min. The cells were washed again with 10 mL of RPMI-1640 medium and resuspended in RPMI-1640 medium supplemented with antibiotics (100 μg/mL penicillin and 100 μg/mL streptomycin). Finally, the cell titers were adjusted to 5 × 10^6^ cells/mL.

### 4.9. Determination of Antigen-Specific Effector Memory T cells

The ability of the studied recombinant proteins to induce the formation of specific CD4+ and CD8+ effector memory T lymphocytes producing cytokines in the spleen was determined. Multiparametric flow cytometry was performed according to the BD Pharmingen^TM^ protocol (BD Biosciences).

Cells were activated in flat-bottomed plates with 10 μg/10^6^ cells of peptides (6 h at 37 °C, 5% CO_2_) or with 1 μg/10^6^ cells of influenza virus A/Aichi/2/68 (H3N2) (24 h at 37 °C, 5% CO_2_) in the presence of 1 μg/mL of Brefeldin A (BD Bioscience, USA) (6 h) and purified anti-mouse CD28 antibodies (24 h). After stimulation, the cells were transferred to plates with a conical bottom to minimize cell loss.

The Fc receptors were blocked with CD16/CD32 antibodies (Mouse BD Fc Block, BD Biosciences) for 30 min. Then, the cells were incubated with Zombie Aqua (Zombie Aqua™ Fixable Viability Kit, Biolegend, San Diego, CA, USA) to detect live cells and subsequently with surface-stained antibodies CD3a-FITC, CD4 PerCP, CD8-APC-Cy7, CD62L-PE-Cy7, and CD44-APC (BD Pharmingen, USA) at 2–8 °C for 30 min.

The cells were permeabilized according to the Cytofix/Cytoperm Plus test system protocol (BD Bioscience, USA) and stained intracellularly with TNF-β-BV421, IFN-γ-PE, and IL-2-BV711 antibodies (BD Biosciences). Data collection (on 100,000 live CD3+ lymphocytes) was performed on a Cytoflex flow cytometer (Beckman Coulter, Brea, CA, USA).

### 4.10. Influenza Virus and Challenge Experiment

Mouse-adapted A/Aichi/2/68 (H3N2) influenza A virus obtained from the Collection of Influenza and Acute Respiratory Viruses at the Research Institute of Influenza was used to challenge the immunized mice at a dose of 4 × LD_50_. The virus was administered intranasally two weeks after the third immunization; the survival and weight of the mice after the challenge were recorded daily for 14 days. Experimental work with influenza strains was carried out in a BSL2 facility. 

The study was carried out according to the Russian Guidelines for the Care and Use of Laboratory Animals. The protocol was approved by the Committee for Ethics of Animal Experimentation at the Research Institute of Influenza (Permit ID 13/a, approved 21 October 2019). All possible efforts were made to minimize the suffering of the animals. 

### 4.11. Statistical Analysis

To compare the groups, the Student’s test (*t*-test) was used. An analysis of flow cytometry data was performed using Kaluza v.2.2 software (Beckman Coulter, Brea, CA, USA). Differences in antigen-specific cytokine-producing T cells were evaluated using the Student’s test (*t*-test) and the Holm–Sidak method for multiple comparisons. All data were checked for normal distribution using the Shapiro–Wilk test. If the *p* value was less than 0.05, the difference was significant. The analysis was carried out by using GraphPad Prism v. 9.5.1. A graphical presentation of the innate immunity and specific T cell response data were produced in the form of Tukey plots, which display the median, lower, and upper quartiles as well as the minimum and maximum values of the sample and outliers.

## 5. Conclusions

We obtained recombinant virus-like particles formed by an artificial self-assembling peptide carrying two epitopes from NP and tandem copies of M2e and HA2 peptides along with the T helper epitope PADRE. Immunization of mice with these particles induced a strong humoral immune response against M2e and the entire virus and led to the formation of cytokine-secreting antigen-specific CD4+ and CD8+ effector memory T cells. Immunization provided high protection of mice against the lethal challenge with influenza A virus. The obtained self-assembling nanoparticles, carrying conserved peptides from M2, HA, and NP proteins of the influenza A virus, as well as the universal T helper epitope PADRE, can be the basis for the development of an influenza vaccine with a wide spectrum of protection.

## Figures and Tables

**Table 1:** Fractions of antigen-specific Tem cells in spleen of immunized mice.

Group of Mice	M2e-Specific Tem Cells		Virus-Specific Tem Cells	
	CD4+	CD8+	CD4+	CD8+
Experimental	0.28 ± 0.07%	0.276 ± 0.11%	5.98 ± 0.63%	13.69 ± 1.74%
Control (PBS)	0.04 ± 0.02%	0.08 ± 0.05%	4.37 ± 0.63%	8.60 ± 1.00%

## Data Availability

The data presented in this study are contained within the article.

## Sample Availability

Not available.

## References

[1] Gerhard W., Mozdzanowska K., Zharikova D. (2006). Prospects for Universal Influenza Virus Vaccine. Emerg. Infect. Dis..

[2] Palese P., García-Sastre A. (2002). Influenza Vaccines: Present and Future. J. Clin. Investig..

[3] Saelens X. (2019). The Role of Matrix Protein 2 Ectodomain in the Development of Universal Influenza Vaccines. J. Infect. Dis..

[4] Schotsaert M., De Filette M., Fiers W., Saelens X. (2009). Universal M2 Ectodomain-Based Influenza A Vaccines: Preclinical and Clinical Developments. Expert Rev. Vaccines.

[5] Jegerlehner A., Schmitz N., Storni T., Bachmann M.F. (2004). Influenza A Vaccine Based on the Extracellular Domain of M2: Weak Protection Mediated via Antibody-Dependent NK Cell Activity. J. Immunol..

[6] El Bakkouri K., Descamps F., De Filette M., Smet A., Festjens E., Birkett A., Van Rooijen N., Verbeek S., Fiers W., Saelens X. (2011). Universal Vaccine Based on Ectodomain of Matrix Protein 2 of Influenza A: Fc Receptors and Alveolar Macrophages Mediate Protection. J. Immunol..

[7] Corti D., Voss J., Gamblin S.J., Codoni G., Macagno A., Jarrossay D., Vachieri S.G., Pinna D., Minola A., Vanzetta F. (2011). A Neutralizing Antibody Selected from Plasma Cells That Binds to Group 1 and Group 2 Influenza A Hemagglutinins. Science.

[8] Tsybalova L.M., Stepanova L.A., Shuklina M.A., Mardanova E.S., Kotlyarov R.Y., Potapchuk M.V., Petrov S.A., Blokhina E.A., Ravin N.V. (2018). Combination of M2e Peptide with Stalk HA Epitopes of Influenza A Virus Enhances Protective Properties of Recombinant Vaccine. PLoS ONE.

[9] Stepanova L.A., Mardanova E.S., Shuklina M.A., Blokhina E.A., Kotlyarov R.Y., Potapchuk M.V., Kovaleva A.A., Vidyaeva I.G., Korotkov A.V., Eletskaya E.I. (2018). Flagellin-Fused Protein Targeting M2e and HA2 Induces Potent Humoral and T-Cell Responses and Protects Mice against Various Influenza Viruses a Subtypes. J. Biomed. Sci..

[10] Jegaskanda S., Job E.R., Kramski M., Laurie K., Isitman G., de Rose R., Winnall W.R., Stratov I., Brooks A.G., Reading P.C. (2013). Cross-Reactive Influenza-Specific Antibody-Dependent Cellular Cytotoxicity Antibodies in the Absence of Neutralizing Antibodies. J. Immunol..

[11] DiLillo D.J., Tan G.S., Palese P., Ravetch J.V. (2014). Broadly Neutralizing Hemagglutinin Stalk-Specific Antibodies Require FcγR Interactions for Protection against Influenza Virus in Vivo. Nat. Med..

[12] Epstein S.L. (2018). Universal Influenza Vaccines: Progress in Achieving Broad Cross-Protection In Vivo. Am. J. Epidemiol..

[13] Shu L.L., Bean W.J., Webster R.G. (1993). Analysis of the Evolution and Variation of the Human Influenza A Virus Nucleoprotein Gene from 1933 to 1990. J. Virol..

[14] Yewdell J.W., Bennink J.R., Smith G.L., Moss B. (1985). Influenza A Virus Nucleoprotein Is a Major Target Antigen for Cross-Reactive Anti-Influenza A Virus Cytotoxic T Lymphocytes. Proc. Natl. Acad. Sci. USA.

[15] Townsend A.R.M., Gotch F.M., Davey J. (1985). Cytotoxic T Cells Recognize Fragments of the Influenza Nucleoprotein. Cell.

[16] Brown L.E., Kelso A. (2009). Prospects for an Influenza Vaccine That Induces Cross-protective Cytotoxic T Lymphocytes. Immunol. Cell. Biol..

[17] Romeli S., Hassan S.S., Yap W.B. (2020). Multi-Epitope Peptide-Based and Vaccinia-Based Universal Influenza Vaccine Candidates Subjected to Clinical Trials. MJMS.

[18] Van Doorn E., Liu H., Ben-Yedidia T., Hassin S., Visontai I., Norley S., Frijlink H.W., Hak E. (2017). Evaluating the Immunogenicity and Safety of a BiondVax-Developed Universal Influenza Vaccine (Multimeric-001) Either as a Standalone Vaccine or as a Primer to H5N1 Influenza Vaccine: Phase IIb Study Protocol. Medicine.

[19] Pleguezuelos O., Robinson S., Stoloff G.A., Caparrós-Wanderley W. (2012). Synthetic Influenza Vaccine (FLU-v) Stimulates Cell Mediated Immunity in a Double-Blind, Randomised, Placebo-Controlled Phase I Trial. Vaccine.

[20] Burkhard P., Meier M., Lustig A. (2000). Design of a Minimal Protein Oligomerization Domain by a Structural Approach. Protein Sci..

[21] Doll T.A.P.F., Dey R., Burkhard P. (2015). Design and Optimization of Peptide Nanoparticles. J. Nanobiotechnol..

[22] Pimentel T.A.P.F., Yan Z., Jeffers S.A., Holmes K.V., Hodges R.S., Burkhard P. (2009). Peptide Nanoparticles as Novel Immunogens: Design and Analysis of a Prototypic Severe Acute Respiratory Syndrome Vaccine. Chem. Biol. Drug Des..

[23] Kaba S.A., Brando C., Guo Q., Mittelholzer C., Raman S., Tropel D., Aebi U., Burkhard P., Lanar D.E. (2009). A Nonadjuvanted Polypeptide Nanoparticle Vaccine Confers Long-Lasting Protection against Rodent Malaria. J. Immunol..

[24] Wahome N., Pfeiffer T., Ambiel I., Yang Y., Keppler O.T., Bosch V., Burkhard P. (2012). Conformation-Specific Display of 4E10 and 2F5 Epitopes on Self-Assembling Protein Nanoparticles as a Potential HIV Vaccine: MPER-Specific Protein Nanoparticle HIV Vaccine. Chem. Biol. Drug Des..

[25] Babapoor S., Neef T., Mittelholzer C., Girshick T., Garmendia A., Shang H., Khan M.I., Burkhard P. (2011). A Novel Vaccine Using Nanoparticle Platform to Present Immunogenic M2e against Avian Influenza Infection. Influenza Res. Treat..

[26] Raman S., Machaidze G., Lustig A., Aebi U., Burkhard P. (2006). Structure-Based Design of Peptides That Self-Assemble into Regular Polyhedral Nanoparticles. Nanomedicine.

[27] Zykova A.A., Blokhina E.A., Stepanova L.A., Shuklina M.A., Tsybalova L.M., Kuprianov V.V., Ravin N.V. (2022). Nanoparticles Based on Artificial Self-Assembling Peptide and Displaying M2e Peptide and Stalk HA Epitopes of Influenza A Virus Induce Potent Humoral and T-Cell Responses and Protect against the Viral Infection. Nanomedicine.

[28] Alexander J., Del Guercio M.-F., Maewal A., Qiao L., Fikes J., Chesnut R.W., Paulson J., Bundle D.R., DeFrees S., Sette A. (2000). Linear PADRE T Helper Epitope and Carbohydrate B Cell Epitope Conjugates Induce Specific High Titer IgG Antibody Responses. J. Immunol..

[29] Ravin N.V., Blokhina E.A., Kuprianov V.V., Stepanova L.A., Shaldjan A.A., Kovaleva A.A., Tsybalova L.M., Skryabin K.G. (2015). Development of a Candidate Influenza Vaccine Based on Virus-like Particles Displaying Influenza M2e Peptide into the Immunodominant Loop Region of Hepatitis B Core Antigen: Insertion of Multiple Copies of M2e Increases Immunogenicity and Protective Efficiency. Vaccine.

[30] Jennings G.T., Bachmann M.F. (2008). The Coming of Age of Virus-like Particle Vaccines. Biol. Chem..

[31] Al-Halifa S., Gauthier L., Arpin D., Bourgault S., Archambault D. (2019). Nanoparticle-Based Vaccines Against Respiratory Viruses. Front. Immunol..

[32] Huber V.C., Lynch J.M., Bucher D.J., Le J., Metzger D.W. (2001). Fc Receptor-Mediated Phagocytosis Makes a Significant Contribution to Clearance of Influenza Virus Infections. J. Immunol..

[33] Jegaskanda S., Vanderven H.A., Wheatley A.K., Kent S.J. (2017). Fc or Not Fc; That Is the Question: Antibody Fc-Receptor Interactions Are Key to Universal Influenza Vaccine Design. Hum. Vaccin. Immunother..

[34] Wohlbold T.J., Chromikova V., Tan G.S., Meade P., Amanat F., Comella P., Hirsh A., Krammer F. (2016). Hemagglutinin Stalk- and Neuraminidase-Specific Monoclonal Antibodies Protect against Lethal H10N8 Influenza Virus Infection in Mice. J. Virol..

[35] Chen Y.-Q., Lan L.Y.-L., Huang M., Henry C., Wilson P.C. (2019). Hemagglutinin Stalk-Reactive Antibodies Interfere with Influenza Virus Neuraminidase Activity by Steric Hindrance. J. Virol..

[36] Bender B.S., Croghan T., Zhang L., Small P.A. (1992). Transgenic Mice Lacking Class I Major Histocompatibility Complex-Restricted T Cells Have Delayed Viral Clearance and Increased Mortality after Influenza Virus Challenge. J. Exp. Med..

[37] Doherty P.C., Topham D.J., Tripp R.A., Cardin R.D., Brooks J.W., Stevenson P.G. (1997). Effector CD4+ and CD8+ T-Cell Mechanisms in the Control of Respiratory Virus Infections. Immunol. Rev..

[38] Gerhard W., Burton D.R. (2001). The Role of the Antibody Response in Influenza Virus Infection. Antibodies in Viral Infection.

[39] Thomas P.G., Keating R., Hulse-Post D.J., Doherty P.C. (2006). Cell-Mediated Protection in Influenza Infection. Emerg. Infect. Dis..

[40] Chalifour A., Jeannin P., Gauchat J.-F., Blaecke A., Malissard M., N’Guyen T., Thieblemont N., Delneste Y. (2004). Direct Bacterial Protein PAMP Recognition by Human NK Cells Involves TLRs and Triggers α-Defensin Production. Blood.

[41] Kannan K., Stewart R.M., Bounds W., Carlsson S.R., Fukuda M., Betzing K.W., Holcombe R.F. (1996). Lysosome-Associated Membrane Proteins h-LAMP1 (CD107a) and h-LAMP2 (CD107b) Are Activation-Dependent Cell Surface Glycoproteins in Human Peripheral Blood Mononuclear Cells Which Mediate Cell Adhesion to Vascular Endothelium. Cell. Immunol..

[42] Clemens E., Van De Sandt C., Wong S., Wakim L., Valkenburg S. (2018). Harnessing the Power of T Cells: The Promising Hope for a Universal Influenza Vaccine. Vaccines.

[43] Sridhar S. (2016). Heterosubtypic T-Cell Immunity to Influenza in Humans: Challenges for Universal T-Cell Influenza Vaccines. Front. Immunol..

[44] De Filette M., Min Jou W., Birkett A., Lyons K., Schultz B., Tonkyro A., Resch S., Fiers W. (2005). Universal Influenza A Vaccine: Optimization of M2-Based Constructs. Virology.

[45] Stoloff G.A., Caparros-Wanderley W. (2007). Synthetic Multi-Epitope Peptides Identified in Silico Induce Protective Immunity against Multiple Influenza Serotypes. Eur. J. Immunol..

[46] Atsmon J., Kate-Ilovitz E., Shaikevich D., Singer Y., Volokhov I., Haim K.Y., Ben-Yedidia T. (2012). Safety and Immunogenicity of Multimeric-001—A Novel Universal Influenza Vaccine. J. Clin. Immunol..

[47] Arai R., Ueda H., Kitayama A., Kamiya N., Nagamune T. (2001). Design of the Linkers Which Effectively Separate Domains of a Bifunctional Fusion Protein. Protein Eng. Des. Sel..

[48] Robinson C.R., Sauer R.T. (1998). Optimizing the Stability of Single-Chain Proteins by Linker Length and Composition Mutagenesis. Proc. Natl. Acad. Sci. USA.

